# A study on the content of integrity policies and research integrity management in Chinese universities

**DOI:** 10.3389/frma.2023.943228

**Published:** 2023-02-10

**Authors:** Yuan Cao, Yuwei Jiang, Yong Zhao

**Affiliations:** Library of China Agricultural University, Beijing, China

**Keywords:** quantitative study, research integrity, academic misconduct policy, Chinese university, rules and regulations

## Abstract

**Background:**

This study outlines a comprehensive analysis of the primary characteristics of managing research integrity (RI) in domestic colleges and universities in China. RI education in China consists primarily of soft advocacy, with no hard requirements or continuous and systematic support. Together with other stakeholders, such as funders and publishers, higher education institutions (e.g., colleges and universities) are one of the vital actors that have a lot of influence on RI promotion and implementation among researchers. However, the literature on the regulation of RI policies in China's universities is limited.

**Methods:**

We investigate the top 50 colleges and universities in the 2021 Best Chinese Universities Ranking. Their guidance and policy documents on RI were collected via their official websites. By integrating the use of scientometrics analysis, including descriptive statistical analysis, inductive content analysis, and quantitative analysis, we examine whether and how these higher education institutions respond to national policies in a timely manner, especially in terms of their frequency of updates, topic clustering analysis, terms clustering analysis, content aggregation. To further understand the composition mechanism and the main working systems of university RI management organizations, we conducted in-depth research on the organizational functions, meeting system, staff composition mechanism, and scientific research misconduct acceptance and investigation mechanisms.

**Results:**

The regulations on the treatment of RI in China's universities have, in response to the government's call to establish their own management policies and working mechanisms, maintained a zero-tolerance stance on research misconduct. The sampled universities listed the definition and principles of misconduct practices, investigation procedures, and sanctions of research misconduct in their own policy documents. Some of them listed inappropriate research practices All 50 sampled universities have formed relevant organizations responsible for RI management, they all provide the detailed regulations of the committees. Yet, there is still a need to further define Questionable Research Practice, foster higher standards for integrity in research and, establish and improve an efficient, authoritative, well-restrained and supervision working mechanism for organizations responsible for RI treatment.

## 1. Introduction

Research integrity (RI) is the cornerstone of scientific innovation, which, in China, is connected to the rate of national innovation development and is an essential aspect of the social integrity system. The academic decisions of colleges and universities are often strongly influenced or even shaped by the government and some other external forces under the hierarchical and “top-down” governance structure in China (Li and Cornelis, 2020). The general offices of the Communist Party of China Central Committee and the State Council issued guidelines supporting the development of the social credit system (The general offices of the Communist Party of China Central Committee the State Council., 2022), which call for strengthening the integrity of scientific research and protecting intellectual property rights, cracking down on thesis trading, and optimizing the protection of (and application system for) intellectual property rights. The prevalence of research misconduct in China in recent years has piqued scholarly and government interest in RI. For example, in May 2018, the General Office of the Central Committee of the Communist Party of China and the General Office of the State Council of the People's Republic of China jointly issued the document “Several Opinions on Further Strengthening the Construction of Research Integrity,” which provides important instructions for improving the management mechanism and responsibility system of RI. According to the report (The State Council The People's Republic of China, 2018):

“All kinds of research institutions, along with those taking part in science and technology management services, must carry out the primary obligation of ensuring research integrity. All kinds of enterprises, institutions, and social organizations engaged in scientific research activities are the primary responsibility for the implementation of research integrity and should make specific arrangements to strengthen research integrity implementation and normalize research integrity management and regulation.”

RI is a critical component of social integrity globally, as well as social norms, especially professional standards for responsible research, which in turn constrain RI. Irresponsible behavior can adversely impact research in at least four ways: (1) undermine the reliability of the research record, (2) weaken the trust colleagues have in one another and the trust the public has in researchers, (3) waste of research funds, and (4) lead to decisions that cause public and/or personal harm (Steneck, 2006). The ethics of research involving human beings is extremely relevant, especially with respect to vulnerable individuals or groups (Novaes and Guilhem, 2017). Fostering Integrity in Research identifies best scientific practices and recommends practical options for discouraging and addressing research misconduct and detrimental research practices (National Academies of Sciences, Engineering, and Medicine, 2017).

Most Chinese universities have their own guidelines focusing on RI (Yi et al., 2019). Previous studies have discussed the differences of specific practices and principles between university groups by comparing them with documents from European universities (Yi et al., 2019) to determine how professional norms are specified in guidance and policy documents on RI in China. The issue of questionable research practices (QRPs) in China has also been investigated (Chi, 2015). Universities can effectively promote the quality of RI by enhancing research management regulations (Zhang, 2014), refining fund management regulations (Jiang, 2017), improving the supervision mechanism of research assessments (Cao et al., 2014), and constructing an RI education system (Zhang and Li, 2019). Meanwhile, many universities have paid attention to the prevention of (and controversy over) abuses of academic power (Ni and Liu, 2017) and have built professional and high-quality scientific research service teams to supervise the implementation of RI (Wang, 2018c). Some studies have analyzed RI education in Chinese universities and discussed the status quo of RI management organizations, objects of teaching, teaching methods, course content, and case publicity education (Yuan, 2019). Others have described the objects, subjects, and nature of courses in RI education in these institutions (Wang, 2019a). Moreover, studies also have proposed building a “one-element, two-level, and three-sided” value system for RI education, as well as establishing a central-network organizational framework (Luo, 2020).

The evaluation of RI policy is an internationally common method that is often done in other countries with a long tradition of RI. CHPS Consulting (2000) undertook a thorough content analysis of research misconduct and RI policies. Ann Lind (2005) evaluated the accessibility and usefulness of research misconduct policies at the top 25 universities ranked by the National Institutes of Health and the National Science Foundation. Harvard University has an RI administrative policy that draws significant attention; it is characterized by a two-level governance framework involving faculty and student management, as well as a combination of prevention and post-event treatment options (Cui et al., 2020). Some studies have examined the RI policies, management institutions, and research misconduct investigations of the University of Minnesota (Liu, 2015), University of Dusseldorf (Wang, 2018a), Aarhus University (Wang, 2018b), Durham University (Wang, 2019b), and Macquarie University in Australia (Zhou, 2019). These standards have been widely discussed and have provided real-world experiences for RI study and management in Chinese universities.

Colleges and universities, as the primary venues for scientific research, are not only vital for talent development but also for shaping researchers and their behavior because research organizations usually have the power to implement certain policies or adopt certain procedures (DuBois, 2010; Anderson, 2018; Bouter, 2018, 2020, Forsberg et al., 2018; Biagioli et al., 2019). Responsibility for ethical research lies with everyone who is active in research, but especially with leaders in research institutions. Researchers' morals alone cannot ensure research integrity; good conditions for exercising integrity must also be created at the level of the organization and the research system (Bouter, 2020). Although many published works present opinions about why RI is important—describing some of the problem areas and how they arose, as well as addressing how RI might be promoted and the possible effectiveness of such efforts—few have presented descriptive statistical analyses and displayed the results visually. Further, the importance of establishing policy for organizations responsible for RI to supervise and govern themselves should not be overlooked. Such investigations do not yet comprise the bulk of the available literature.

How organizations work with their RI and legitimacy is something that is embedded in routines and practices, and thus part of everyday organizational life. The White House Office of Science and Technology Policy (OSTP) stated that agencies need to strengthen scientific integrity policies to deter undue influence in the conduct, management, communication, and use of science; that violations involving high-level officials are the most problematic and difficult to address; and that further action is required to establish and maintain a culture of scientific integrity across all individuals and agencies that conduct, manage, communicate, and make use of science (National Science Technology Council, 2022). In the words of Becker (1998) and European Commission (2020), organizational RI requires ongoing action. Paine (1994) has pointed out convincingly that RI can be managed. This enables us to move our understanding toward the work undertaken to prevent it from happening in the future. To capture these dimensions, we think that a concept of “RI management” is useful. It can be described as the ongoing organizational activities and strategies associated with developing, repairing, assessing and/or maintaining RI. RI management can be further defined particularly as “a kind of supervise assessment on scientific integrity policies of departments and agencies and instances in which they have not been followed or enforced, and it identifies effective practices for strengthening scientific integrity in specific areas, including training and transparency in scientific integrity, handling scientific disagreements, supporting professional development of scientists, addressing emerging challenges to scientific integrity, and effective communication of the results of organizational scientific activities.

Therefore, we reviewed documents on RI policy across a wide range of Chinese universities to present a descriptive statistical analysis with visualization of the overall situation and main characteristics of RI regulations. We explore how their RI-management organization supervises and regulates themselves with the aim to inform national RI implementation and development in China. The purpose of this work is to summarize the effectiveness and shortcomings of RI management in China's universities and to provide a decision basis and policy reference for further strengthening the implementation of RI in China.

## 2. Data processing and study design

### 2.1. Data processing

#### 2.1.1. Sampled university selection

Most colleges and universities in China have promulgated policies to clarify the content and requirements of RI. They have set up a management or advisory office to carry out the specific management work of RI and to formulate norms and systems (Yi et al., 2019). Therefore, in this study, we took the top 50 universities in China, as ranked by the 2021 Best Chinese Universities Ranking (Ranking, 2021), as the research sample.

#### 2.1.2. Document selection

##### 2.1.2.1. Inclusion criteria

According to the research purpose of our study, the universities and colleges' policies were included only if

a. Documents were handling the research integrity/misconduct, or the procedure for dealing with it.
b. Documents targeted the organization(s) responsible for RI treatment, academic/standardized/special committee(s) and other format of supervisory bodies dealing with research integrity/misconduct.

##### 2.1.2.2. Exclusion criteria

Documents that mainly conveyed activity of teaching and studying were not considered.

#### 2.1.3. Search approach

We searched for documents by applying two approaches:

a. Main: We obtained available relevant policy documents related to RI and organizations responsible for RI treatment from the universities' official websites including the information disclosure page and academic committee page.
b. Supplementary: Additional documents and relevant news reports mentioned in our collected documents were obtained and supplemented by searching the mentioned titles/names in the search engine.

The following keywords and subject words were used in this step: “学风建设(construction of academic atmosphere),” “科研诚信 (Research Integrity),” “学术不端(academic scientific misconduct),” “科研不端(research misconduct),” “学术道德(academic morality),” “捏造/造假(fabrication),” “篡改(falsification),” “剽窃/抄袭(plagiarism),” “一稿多发(multiple publication).” The document searches and all analysis were conducted in April 2022.

### 2.2. Study design

#### 2.2.1. Text preprocessing

The text preprocessing was divided into two main parts.

For the documents related to RI treatment management, the following steps were included:

First, we standardized the data: after obtaining the documents related to RI treatment management and organizations responsible for RI treatment, we recorded the promulgation organization, policy name/title, promulgation time, definition of research misconduct, policy access steps, web links and the accessibility to the documents (details see Supplementary Tables 1A, B). Second, to enhance the accuracy of the subject word classification results, we developed a self-built categorization matrix composed of relevant subject words based on the reading and understanding of policies and general pattern related to RI. Third, splitting words. Combining the categorization matrix built by ourselves, the word bank of HIT, Discontinued Word Bank of Baidu, and a Chinese splitting software named jiebaR were used to split the words of the policy text and eliminate the words with no practical meaning such as prepositions and adjectives. Finally, results obtained from step three are de-duplicated, vectorized and the keywords of each policy text were extracted using Word Frequency–Inverse Document Frequency (TF-TDF).

For the documents related to organizations responsible for RI treatment, we recorded the general information of the research integrity management organization, such as organization name, competent authorities, whether promulgated regulations, web links and other necessary information (see supporting materials).

#### 2.2.2. Classification and coding

A inductive content analysis (Elo and Kyngäs, 2008; Elo et al., 2014) was followed by this study. First, to understand the nature of the collected data, content and general pattern, all documents were carefully read thrice by 3 researchers with background in library intelligence and the preliminary observations were discussed among them. Second, to organize the categorization matrix of RI policy was developed according to the code and rules for subject word extraction from collected policy documents. Third, all extracted subject words were coded in accordance with the categorization matrix, after continuous feedback and refinement in the context labeling, and examples of the code and rules for extraction can be find in Supplementary Table 1.

#### 2.2.3. Content labeling and reliability testing

**Figure 1:**
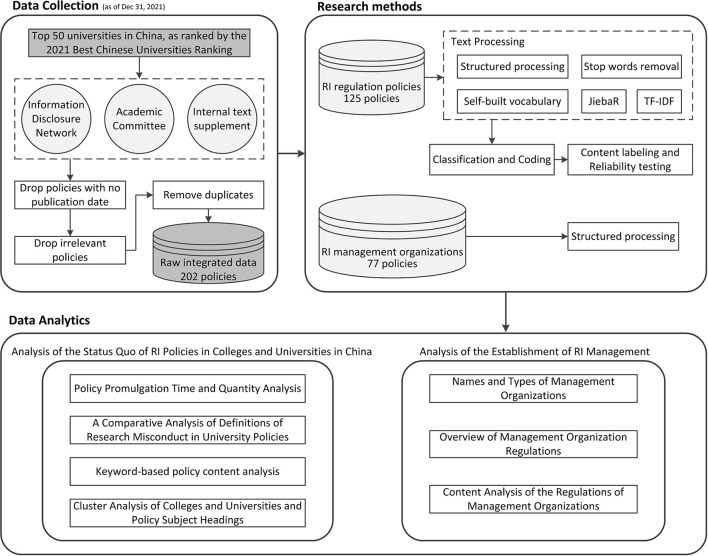
The flowchart illustrating the data processing in this study.

The coding process was developed independently by three coders with backgrounds in library intelligence. First, 20 policies were randomly selected, and the policy content was manually annotated according to the categorization matrix. During the annotation process, the text describing the specific topic/theme in the policies was color-coded to facilitate the coders' later cross-checking. For inconsistent parts of the annotation, catalogers reached agreement through discussion and meanwhile improved the categorization matrix. Second, coders labeled the remaining 105 policies according to the categorization matrix coding rules. Third, when the labeling process was completed, the consistency of the labeling results was checked using Krippendorff alpha reliability analysis (Krippendorff, 2018), and the test result showed that the alpha coefficient was 0.872. According to Krippendorff's reliability test, an alpha > 0.8 indicates a high level of credibility results, therefore the content labeling results of this paper have a high credibility. Finally, again the coders finalized the consistent content annotation results by discussion. The overall data processing of this paper is shown in Figure 1.

## 3. Results

### 3.1. Analysis of the status quo of RI policies in colleges and universities in China

#### 3.1.1. Policy promulgation time and quantity analysis

##### 3.1.1.1. Number of policies

**Figure 2:**
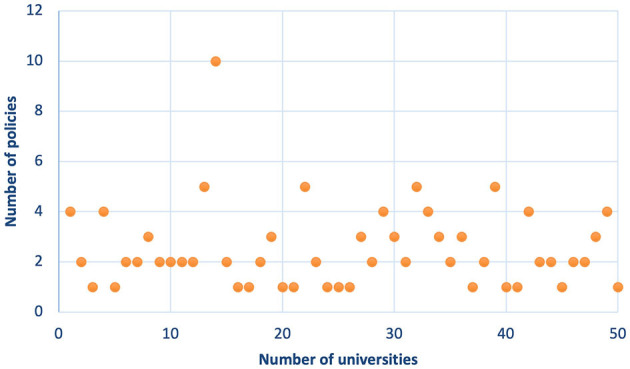
Distribution of the number of university RI policies.

By analyzing the policy distribution characteristics, policy changes, policy diffusion, policy themes, and other factors, these measurements provided researchers with empirical data and objective descriptions of policies (Li et al., 2015). From the distribution of university RI policies (Figure 2), it is evident that all universities in the sample have formulated university-level RI policies; there is a total of 125 policies and an average of 2.5 policies per university. Further, 34 universities have promulgated multiple policies to regulate RI management, among which 18 universities have established more than or equal to three RI policies. They have also formulated regulations for RI management from different perspectives thus developing more comprehensive regulations. For example, Sichuan University established 10 RI policies, and Beijing Normal University, Hunan University, and Lanzhou University have each developed five RI policies.

##### 3.1.1.2. Time distribution

**Figure 3:**
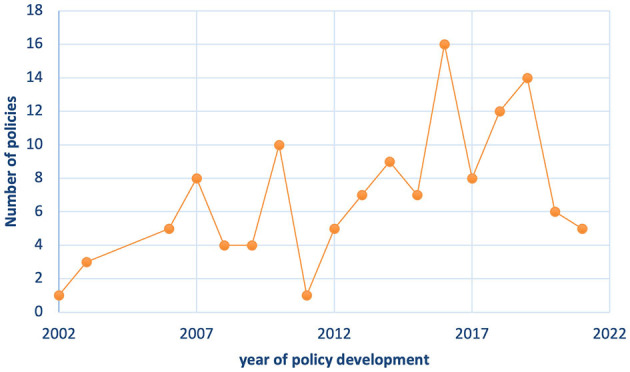
Distribution of the time of promulgation of RI policies in universities. The number RI policies developed/and implemented throughout the years. The x-axis shows the year of development/implementation, and the y-axis shows the number of policies.

Universities began writing RI policies in 2002 (Figure 3), with clear upward trends in policy development occurring in 2015 (The general offices of the Communist Party of China Central Committee the State Council., 2015) and 2018 (The general offices of the Communist Party of China Central Committee the State Council, 2018). Notably, in 2015, the report “Opinions on Deepening the Reform of Institutional Mechanisms and Accelerating the Implementation of Innovation-Driven Development Strategy” issued by the general offices of the Communist Party of China Central Committee and the State Council proposed the development of innovation performance evaluation methods of scientific research institutions and innovation policy coordination review systems. In 2018, another report, “Opinions on Deepening the Reform of Project Evaluation, Talent Evaluation, and Institutional Assessment,” established “a classification evaluation system oriented to the quality, contribution, and performance of scientific and technological innovation”; that is, China's science and technology evaluation practices have entered a new historical stage. To a certain extent, this indicates that the national government provides guidance at the macro level, while local universities follow up with implementations at the micro level, such as system updates. To summarize, the number of policies promulgated by the sample universities each year shows an overall trend of fluctuating growth, with a strong increasing trend in recent years.

During this research, we found that at least 22 universities in the sample updated RI policies (Table 1) and completed the revision or update of 29 policies. Among them, Fudan University, Sun Yat-sen University, China Agricultural University, Huazhong Agricultural University, and Harbin Engineering University revised and updated several policies. Since the issuance of the report “Several Opinions on Further Strengthening the Construction of Research Integrity” in May 2018 and “Opinions on Further Promoting Scientists' Spirit and Strengthening the Construction of Work Style and Academic Style” in June 2019, the policies of nine universities (Zhejiang University, Shanghai Jiaotong University, Sun Yat-sen University, Central South University, Xiamen University, China Agricultural University, Xi'an University of Electronic Science and Technology, Northeast Normal University, and Harbin Engineering University) responded to the important RI guidelines issued by the state. This shows that many universities are paying more attention to RI management to ensure that their policies follow changes in the regulatory environment, are in line with the direction of national policies, contain updated forms of RI management, and improve their work plans.

**Table 1:** RI policy turnover in higher education.

	**Name of current policy**	**Current policy implementation time**	**Original policy promulgation time**
Tsinghua University	Measures for the Prevention and Handling of Academic Misconduct at Tsinghua University (Tsinghua University, 2017)	2017	2003
Beijing University	Code of Academic Ethics for Teachers of Peking University (Beijing University, 2007)	2007	2002
Zhejiang University	Rules for Handling Academic Misconduct at Zhejiang University (Zhejiang University, 2018)	2018	2017
Shanghai Jiao Tong University	Academic Code for Graduate Students of Shanghai Jiao Tong University (Shanghai Jiao Tong University, 2019)	2019	2017
Fudan University	Academic Code of Fudan University (Fudan University, 2017a)	2017	2014
	Regulations on the Implementation of the Academic Code of Fudan University (Fudan University, 2017b)	2017	2014
Xi'an Jiaotong University	Xi'an Jiaotong University Academic Code of Conduct and Treatment of Violations (Xi'an Jiaotong University, 2006)	2006	2003
Sun Yat-sen University	Sun Yat-sen University Academic Ethics Guidelines (Sun Yat-sen University, 2020)	2020	2018
	Measures for the Prevention and Handling of Academic Misconduct of Sun Yat-sen University (Sun Yat-sen University, 2019)	2019	2018
Beijing University of Aeronautics and Astronautics	Management Measures for the Use of the “Academic Misconduct Detection System” for Postgraduate Dissertations of Beijing University of Aeronautics and Astronautics (trial implementation) (Beijing University of Aeronautics Astronautics, 2015)	2015	2014
Tianjin University	Academic Standards for Postgraduates of Tianjin University (Tianjin University, 2017)	2017	2013
Central South University	Measures for Preventing and Handling Academic Misconduct of Central South University (Central South University, 2021)	2021	2018
Northwestern Polytechnic University	Academic Ethics Code and Management Measures of Northwestern Polytechnic University (Northwestern Polytechnic University, 2016)	2016	2009
Xiamen University	Measures for Handling Academic Misconduct and Academic Misbehavior of Xiamen University (Xiamen University, 2018)	2018	2015
South China University of Technology	Management Measures of Academic Ethics for Science and Technology Staff of South China University of Technology (South China University of Technology, 2009)	2009	unknown
Dalian University of Technology	Rules for the Prevention and Treatment of Academic Misconduct of Dalian University of Technology (trial implementation) (Dalian University of Technology, 2017)	2017	2014
China Agricultural University	Rules for Investigation and Handling of Research Integrity Cases of China Agricultural University (trial implementation) (China Agricultural University, 2020)	2020	2008
	Rules for Investigation and Handling of Research Integrity Cases at China Agricultural University (trial implementation) (China Agricultural University, 2020)	2020	2017
University of Science and Technology Beijing	Implementation Rules of Academic Style Construction of University of Science and Technology Beijing (University of Science Technology Beijing, 2012)	2012	2010
Northeastern University	Implementation Rules for Investigation and Handling of Academic Misconduct of Northeastern University (revised) (Northeastern University, 2017)	2017	2016
Xi'an University of Electronic Science and Technology	Code of Academic Ethics of Xi'an University of Electronic Science and Technology (revised) (Xi'an University of Electronic Science Technolog, 2019)	2019	2015
Huazhong Agricultural University	Academic Code of Huazhong Agricultural University (revised) (Huazhong Agricultural University, 2014a)	2014	2009
	Interim Measures for Handling Academic Misconduct at Huazhong Agricultural University (Huazhong Agricultural University, 2014b)	2014	unknown
East China University of Science and Technology	Measures for Handling Academic Misconduct and Implementation Rules of East China University of Science and Technology (East China University of Science Technology, 2016)	2016	2012
Northeast Normal University	Academic Misconduct of Postgraduate Students' Dissertations and Handling Measures of Northeast Normal University (Northeast Normal University, 2021)	2021	2016
Harbin Engineering University	Measures for the Construction of Research Integrity and Academic Misconduct of Harbin Engineering University (Harbin Engineering University, 2019)	2019	2002, 2009, 2009, 2012

#### 3.1.2. A comparative analysis of definitions of research misconduct in university policies

Research misconduct is an important aspect of research integrity (Fanelli, 2009; Li, 2011; Bouter, 2015), and its definition is essential to RI management. To carry out RI management, we must first clarify the performance and scope of research misconduct. Therefore, the next step in the research process is to conduct a comparative analysis of definitions of research misconduct in university policies. Our research shows that there are 78 policies defining common research misconduct, and each university clearly specifies the main examples of this behavior. We classify research misconduct into 18 specific manifestations, such as plagiarism and appropriation of academic achievements; fabrication and falsification of data, graphs, or conclusions; alteration of data or research results; and improper attribution. Overall, universities describe research misconduct in their policies in more detail, among which Wuhan University and Zhejiang University outline 15 specific manifestations of research misconduct, with a coverage rate of 83.33%. As shown in Table 2, universities have a highly unified understanding of the basic forms of research misconduct—plagiarism, fabrication, and falsification—and pay more attention to the common methods of research misconduct, such as improper authorship, falsification of academic experience, buying and selling papers, and ghost-writing/ghost-submitting papers. However, some forms of irresponsible behavior, such as abuse of academic reputation, abuse of academic power, disclosure of academic secrets, and misuse of research resources, are not readily identifiable by others; in other words, these abuses are difficult to prove, take much more time to investigate, and are not yet clearly reflected in national policies. Therefore, only a few universities have explicitly prohibited those forms of misbehavior. However, because of the concealment and complexity of this kind of research misconduct, its adverse effects are no less than those of other misdeeds and deserve the attention of RI management. In addition, some instances of research misconduct, such as violations of research ethics and laboratory regulations, are rarely mentioned even though they are essential points of concern for RI management.

**Table 2:** Specific manifestations of research misconduct in the RI policy of higher education institutions.

**Specific manifestations of research misconduct**	**Number of policies**	**Policy proportion**
Plagiarism, and misappropriation of academic achievements	76	97.44%
Fabrication of charts, conclusions, etc.	72	92.31%
Falsification of data or research results	71	91.03%
Improper authorship	66	84.62%
Falsification of academic experience	58	74.36%
Dissertation trading, ghost-writing, ghost-submitting	57	73.08%
Multiple submissions and duplicate publications	36	46.15%
Abuse of academic prestige	28	35.90%
Interfere and obstruct research activities by improper means	25	32.05%
Disclosing academic secrets	23	29.49%
Misuse of research resources	22	28.21%
Authorizing, directing, assisting, or harboring research misconduct	20	25.64%
Abuse of academic power	17	21.79%
Improper citation	15	19.23%
Misappropriation of collective research results	9	11.54%
Violation of research ethics	6	7.69%
Violation of laboratory regulations	4	5.13%
Other research misconduct	70	89.74%

#### 3.1.3. Analysis of policy content

**Figure 4:**
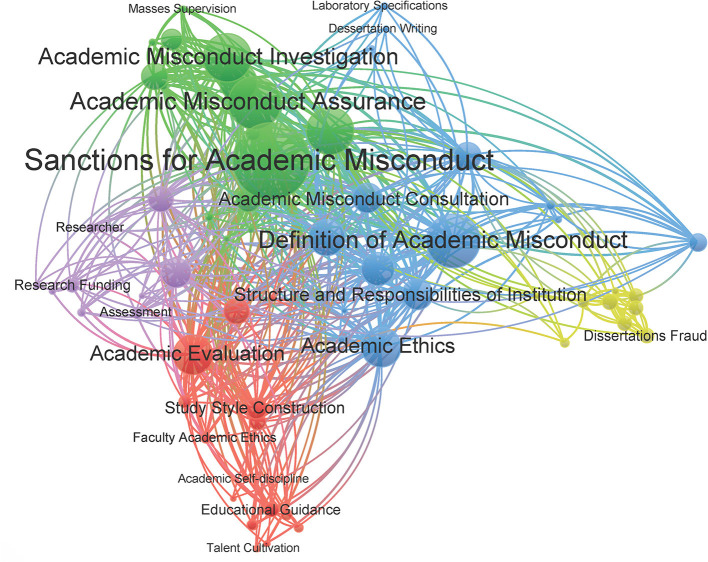
Cluster relation of the keywords of university RI policies.

Policy content analysis can summarize the focus and direction of university RI management. In this study, keywords were extracted using TF-IDF from the original texts of policies from universities in the sample, and the extracted keywords were cleansed to eliminate words with repeated meanings; the high-frequency keywords are shown in Table 3. In addition, this paper uses VOSviewer software to cluster the keywords of university RI policies based on the co-word analysis method (Figure 4).

**Table 3:** High-frequency keywords for RI policy in higher education (word frequency ≥ 9).

**Policy subject headings**	**Frequency**	**Policy subject headings**	**Frequency**
Sanctions for Academic Misconduct	86	Structure and Responsibilities of Institution	20
Definition of Academic Misconduct	53	Project Declaration	19
Academic Misconduct Assurance	43	Academic Misconduct Consultation	16
Academic Misconduct Investigation	40	Research Integrity Education	16
Appeal and Review	37	Research Data Management	14
Academic Ethics	30	Graduate Students	13
Academic Evaluation	28	Academic Environment	13
Academic Misconduct Reception	26	Study Style Construction	13
Intellectual Property	25	Information Security	13
Normative Citations	24	Dissertation Fraud Sanctions	9
Academic Norms	22	Academic Misconduct Prevention	9

According to the clustering results in Figure 4, this study converges the thematic contents represented by the extracted keywords into five major sub-categories based on manual labeling: (1) RI construction: academic style construction, academic ethics construction, academic evaluation, scientific research data management, academic supervision, and self-regulatory awareness; (2) academic misconduct handling process: academic misconduct acceptance, academic misconduct; (3) definition of academic misconduct and RI: definition of academic misconduct, intellectual property rights, standardized citation, paper publication, paper writing, laboratory standards; (4) the process of handling dissertation falsification: reporting, investigating, and punishing academic dissertation falsification, quality of dissertation, responsible body; and (5) the code of conduct of scientific researchers: research funding, scientific research behavior norms, subject declaration, assessment and evaluation, academic review, education, and publicity.

As illustrated in the results of the high-frequency keyword analysis, RI management in universities has some commonalities. First, all universities can clearly list the manifestations of research misconduct (such as falsification, fabrication, and plagiarism) and have developed clear descriptions of the content of academic norms (such as normative citation, intellectual property rights, subject declaration). This clarifies the basic concept of RI and lays the foundation for RI management work. In particular, seven policies define the violation of human or animal research subject regulations as academic misconduct or the violation of academic norms. Second, most universities have clarified in their policies the serious consequences that will result from research misconduct, and they define the university-level RI management organization and workflow for accepting and investigating research misconduct cases. This step initially clarifies the working ideas of RI management. Some colleges and universities have formulated separate policy documents on the processing steps and regulations related to the falsification of dissertations and further refined the objects of RI management. It is worth mentioning that 13 policy elements clearly state that teachers are jointly and severally liable and punished when lack of supervision leads to misconduct by their students.

On the other hand, RI management in universities still needs to be strengthened. First, RI education and academic misconduct reporting and prevention are important ways to limit these violations. However, these three topics are mentioned infrequently in university RI policies. Second, each university emphasizes the implementation of RI in their policy, but scientific research data management appears only 14 times in these documents. In 2018, after the General Office of the State Council issued the “Measures for the Administration of Scientific Data,” some colleges and universities issued complete and systematically written scientific research data management systems or platforms, but others have not followed up and implemented them. In general, the main problems of RI management have been clarified by various colleges and universities through formulating policies, but some universities need to improve the related work of RI reporting, prevention, education, and data management.

#### 3.1.4. Cluster analysis of colleges and universities and policy subject headings

**Figure 5:**
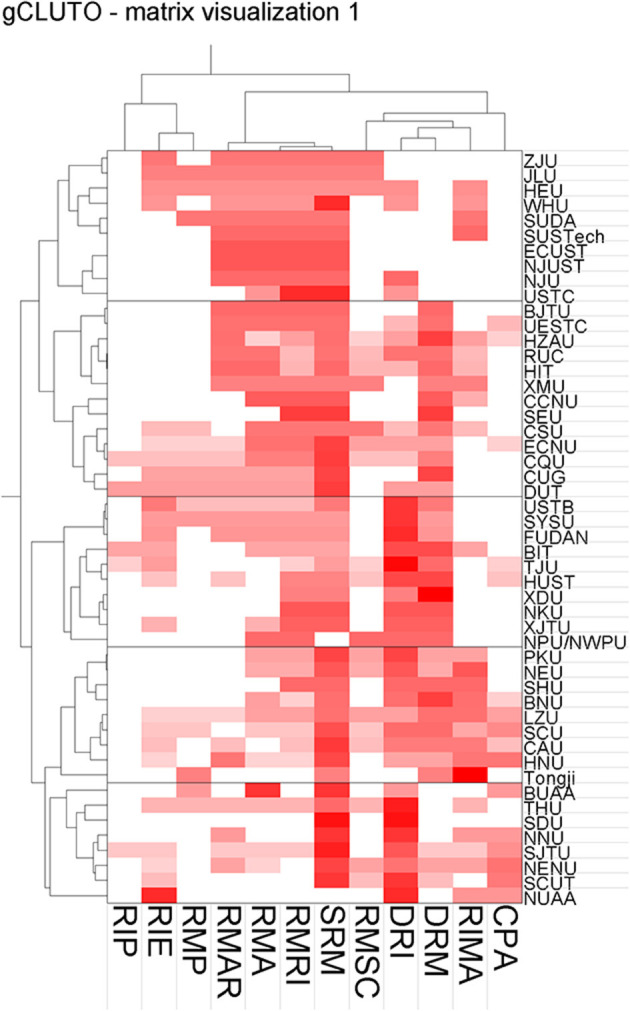
Clustering results of RI policy themes in higher education institutions (CPA, Code of Policy Application; RIMA, Research Integrity Management Agency; DRM, Definition of Research Misconduct; DRI, Definition of Research Integrity; RMSC, Research Misconduct Supervision and Consultation; SRM, Sanctions for Research Misconduct; RMRI, Research Misconduct Reception and Investigation; RMA, Research Misconduct Assurance; RMAR, Research Misconduct Appeals Review; RMP, Research Misconduct Prevention; RIE, Research Integrity Education; RIP, Research Integrity Promotion).

To further analyze the relationship between universities and RI policy topics, this study draws a clustering map of RI policy topics at the sampled universities based on the average cosine similarity among policy subject structures (Figure 5). According to the clustering results, the 50 colleges and universities can be divided into five types. The Y-axis in Figure 5 is 12 RI policy topics, and the horizontal coordinate is the top 50 sample universities. The lattice that crosses the rows and columns represents the weight of the college's publications on the policy subject; the colors, from light to dark, represent the university's emphasis on the subject, with deeper colors signifying greater focus.

The descriptions of the clustering results are shown in Table 4. Isim refers to the average similarity of the cosine, namely, the subjects of university RI policies. Descriptive features refers to the percentage of university subject similarities that the topic can explain within a class. According to Figure 2, the common characteristic of the policy theme in Class 1 is the definition of RI. Distinguishing features refers to the percentage of university subject differences that the topic can explain compared to other university policy topics. For example, Research Misconduct Reception and Investigation (RMRI) can explain 31.1% of the average difference between the policy topics of the first type of university and those of the other colleges and universities. Compared with the policy topics of the first university, other colleges and universities provide relatively little introduction to the continuous investigation of scientific research.

**Table 4:** Characteristics of clustering results of RI policy themes in higher education institutions.

**Cluster**	**Size**	**Isim**	**Descriptive features**	**Discriminating features**
1	8	0.758	DRI (40.5%), SRM (36.1%), CPA (8.8%), RIE (3.8%)	RMRI (31.1%), DRM (22.2%), DRI (20.3%), CPA (13.5%)
2	9	0.838	SRM (28.5%), RIMA (22.4%), DRM (17.6%), DRI (16.2%)	RIMA (50.0%), RMAR (14.8%), RMA (13.1%), RMRI (10.7%)
3	10	0.837	DRI (35.5%), DRM (26.6%), RMRI (13.8%), SRM (13.0%)	DRI (22.5%), SRM (19.4%), RIMA (17.2%), DRM (16.8%)
4	13	0.831	SRM (28.2%), DRM (22.9%), RMRI (16.5%), RMA (5.9%)	DRI (54.1%), DRM (18.9%), RIMA (5.9%), RMA (5.9%)
5	10	0.803	SRM (27.4%), RMRI (23.8%), RMA (20.5%), RMAR (17.4%)	DRM (44.7%), DRI (21.4%), RMAR (13.0%), RMA (8.5%)

Combined with the clustering results according to the code (Table 5), five characteristics of the RI policy themes can be obtained through comparative analysis. The first type of universities is that the content of policies focused on the Definition of Research Integrity (DRI), Sanctions for Research Misconduct (SRM), RMRI, and Definition of Research Misconduct (DRM) are relatively fewer in number; we summarize them as “research misconduct restriction regulatory type.” The second type, content focused on the SRM and Research Integrity Management Agency (RIMA) is relatively balanced with other topics; we classify them as “research integrity system construction type.” The third type, the content focused on the DRI, DRM, and SRM is addressed less frequently; we summarize these topics as “RI definition type.” The fourth type, the topics of SRM, DRM, and DRI are clearly lacking; we summarize them as “scientific research misconduct restraint type.” Finally, the fifth type, the content addressing SRM, RMRI, and DRM is also limited; these are summarized as “scientific research misconduct fine management type.” Generally, the punishment of research misconduct is the significant feature or distinguishing theme of RI policies at the colleges and universities under study.

**Table 5:** Code and rule for extracted the subject category used to describe the RI policies.

**Subjects category**	**Code and rule for extraction**
Policy Application Range	Description of the applicable group, application conditions
Definition of Research Integrity	The policy contains the definition of the RI, academic norms, academic ethics and concepts, and lists the behaviors or manifestations that abide by RI, academic norms, academic ethics and research ethics from the positive introduction of RI sentences, salutes, paragraphs
Definition of Academic Misconduct	The definition and concept of research misconduct appears in the policy, sentences, clauses and paragraphs which introduce RI from the opposite side are listed
Research Integrity Management Organization	The policy describes the management organizations that issues the RI, academic misconduct, study style construction, teacher ethics construction, academic research ethics and other issues
Academic Misconduct Supervision and Reporting	The policy describes the supervision/reporting conditions reporting materials, reporting procedures of academic misconduct
Academic Misconduct Acceptance and Investigation	The policy describes the admissibility investigation related to academic misconduct for investigation procedures, investigation methods, establishment of investigation teams, rules of investigation, presentation of results
Academic Misconduct Assurance	The policy states the conditions of academic misconduct assurance and conditions for determining different levels
Sanctions for Academic Misconduct	The policy describes the punishment for academic misconduct
Appeal and Review for Academic Misconduct	The policy describes the duration, responsible departments and submission procedures of the admissibility appeal and review for academic misconduct when the whistle-blower has objections to the results of the investigation and/or the punishment
Research Integrity Education	The policy points out the establishment of relevant education system or proposes ways and means to promote RI education and other related contents of RI education
Research Integrity Reward	The policy states the conditions and content of rewards for people whit good RI and teacher ethics
Academic Misconduct Prevention	The policy describes the precautions against academic misconduct in scientific research

### 3.2. Analysis of the establishment of RI management

#### 3.2.1. Names and types of management organizations

Universities are the primary institutions responsible for RI, and it is necessary to establish special organizations or working groups to oversee the development of these management processes. Our investigation found that all 50 sampled universities have formed relevant organizations responsible for RI management. These organizations either guide the development and management of RI treatment and plan institutional frameworks, or directly participate in the specific management of RI treatment, which promotes consideration of RI procedures in universities from different perspectives. Upon considering the names of RI management organizations, despite the various types and complicated labels, we divided the offices into two main categories. An academic committee is the first type of organization that handles the main management work of RI treatment. The second type is the specific committee that handles more detailed work of RI-related matters, including academic construction committee which integrates management of RI treatment with academic construction activities, academic standards or ethics which is a type of group that focuses on a certain aspect of RI, such as implementation of academic rules and norms. The academic committee is the highest level of academic authority in research institutions, and it is a basic component of academic management in research institutions (Yuan and Du, 2014). Twenty-five colleges and universities from the sample directly manage their RI through university academic committees, and most of the remaining colleges and universities manage their RI using some other specialized management or advisory organizations housed under university academic committees from which they receive supervision and guidance. Generally, university academic committees coordinate the RI management of each university, and different kinds of special committees play a further management role.

#### 3.2.2. Overview of management organization regulations

**Figure 6:**
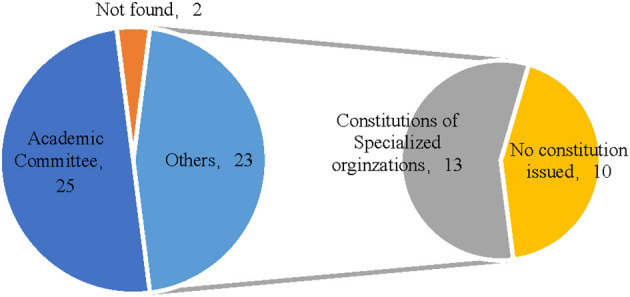
Statistics on the promulgation of RI management organizations and charters in universities.

The regulations laid out by RI management organizations are the fundamental norms to which these bodies must adhere. According to the analysis, university-level academic committees play a coordinating role in RI management; hence, this study first investigated the constitution by-laws of university academic committees and found that 48 of the sampled universities promulgated and disclosed their committee's by-laws in both university-level and institution-level, while Beijing Jiaotong University publicized both but did not disclose them. Thus, almost all the universities under study developed detailed regulations on the authority and responsibility, operation mechanism, and workflow of their RI management organizations. In addition, 23 colleges and universities in the sample established special RI management organizations, among which 13 promulgated and disclosed the constitution or procedures of the special management organizations (Figure 6), further clarified the objects and practices of RI management, and standardized the content and processes of scientific research misconduct reporting, acceptance, investigation, identification, punishment, appeals, and review based on the constitution of the academic committee. A majority of the colleges and universities have completed the development of regulations and systems, and nearly half have completed the institutional regulations specifically for RI work.

#### 3.2.3. Content analysis of the regulations of management organizations

To further analyze the content of regulations that guide university RI management organizations, our study analyzed the full text of the constitutions of 13 specialized committees that were identified in the analysis. The results show that most institutional regulations mainly focus on the nature and orientation of the organization, its development goals, main responsibilities, rules of institutional personnel composition, institutional rights and obligations, and operation mechanisms. To further understand the composition mechanism and the main working systems of university RI management organizations, we conducted in-depth research on the organizational functions, meeting system, staff composition mechanism, and scientific research misconduct acceptance and investigation mechanisms of these institutions. The results of the research show that (Table 6), in terms of organizational functions, RI management offices are mainly responsible for formulating relevant policies, accepting and investigating research misconduct cases, and organizing and taking part in RI education. As for holding meetings, most universities have formed a regular plenary session system in addition to a plenary meeting convened by the director as needed. In terms of tenure requirements, most colleges and universities have decided on the membership size, discipline, title, and position. The term of office of the personnel serving on the specialized RI management bodies is the same as that of the academic committee of the university; at some universities, members can be elected for a second term, but the term of office cannot exceed two terms. As for the investigation mechanism, six universities introduce in detail the whole process of receiving and investigating research misconduct cases, and they stipulate the time limit for the completion of each aspect of the work; the rules and regulations are fully comprehensive. The content is meticulous and complete, which is of great significance for guiding this work. Overall, the statutes of the specialized organizations for RI management provide a basic introduction to their responsibilities, as well as their composition and operation, which can provide guidance for their work, but some office's statutes do not provide enough details on the investigation process, time limit, or appeals process and review, which need to be improved.

**Table 6:** Research on statutes of specialized institutions for RI management in universities.

**Rules and regulation categories**	**Content**	**Number of colleges and universities**	**Specific regulations (number of colleges and universities)**
Organizational functions	Formulation of guidelines and policies	13	None
	Receiving and investigating cases of research misconduct	13	None
	Education on research integrity	13	None
Meeting regulations	Regular plenary session system	13	Once per academic year (2), semi-annually (11)
	Plenary meeting convened by the director	13	None
	Population	12	Convene on demand (12)
Tenure requirements	membership size	13	None
	Academic discipline	13	None
	Title/academic level	13	Experts and scholars (4), positive senior title (6), senior professional and technical personnel (3)
	Post committee members	13	None
Term of office rules	Term of office	13	4 years (11), 5 years (2)
	Reappointment	13	None
	Terms of Re-election	13	Maximum of two terms (13)
	The proportion of re-election	11	No more than 2/3 of the previous members (11)
Research misconduct acceptance and investigation mechanism	Length of response to inquiries	3	5 working days (2), 15 working days (1)
	Initial investigation period	2	15 working days (1), 40 working days (1)
	Time limit for notifying the respondents of the results of the preliminary investigation	1	5 working days (1)
	Time limit for the formation and consideration of the investigation report	3	15 working days (1), 40 working days (1), 60 working days (1)
	Time limit for notifying respondents of the results of processing	2	Informed on the spot (2)
	Time limit for submitting an appeal	6	5 working days (3), 10 working days (1), 30 working days (2)
	Time limit for responding to an appeal	2	15 working days (2)
	Reconsideration period	1	15 working days (1)

## 4. Discussion

Research misconduct is a global concern which is the same as in China. Realizing the importance of RI, Chinese government and universities have also taken measures and published a large number of documents to protect and promote it (Ministry of Science Technology of the People's Republic of China, 2006; Ministry of Education of the People's Republic of China, 2014; The general offices of the Communist Party of China Central Committee the State Council., 2015; The general offices of the Communist Party of China Central Committee the State Council, 2018). The governance structures of Western countries in RI are generally triangular, whereas the one in China is an inverted triangle—the government plays the leading role, and the learned societies and research institutions are relatively weak and only follow the government (Li and Huang, 2017; Li and Cornelis, 2020). The Chinese universities, colleges, and academic committees are highly dependent on the government and its academic decisions are positively keeping highly accordance with the governmental regulation, opinions, guidance, and decisions (Li and Cornelis, 2020), and this structure makes universities, colleges and academic committees subordinates of governmental bodies. The presence of elite universities is an important yardstick for measuring the development level of a country's higher education institutions and a critical factor in the overall strength of a nation's science, technology, and economy. One severe case of research misconduct intensely affects a university's academic reputation, and rebuilding from this can be a slow and difficult process. RI is a necessary form of system security for not only remaining reputation but also ensuring that these institutions are conducting research and producing results that are trustworthy and reliable. Fostering RI through establishing and perfecting a university's integrity policy is mandatory from an international perspective.

This study showed that there is a prominent number of policies and initiatives aiming at RI promotion in Chinese universities. We analyzed the DRM in university RI policies and finds that all sample universities have in response to the government's call to establish their own management policies and working mechanisms, maintained a zero-tolerance stance on research misconduct.

### 4.1. Definition of scientific misconduct

Slightly all of the policies reviewed for our analysis contain a definition of research misconduct that goes beyond the standard definition of research misconduct used by Ministry of Science and Technology of the People's Republic of China (Ministry of Science Technology of the People's Republic of China, 2006), including fabrication, falsification, and plagiarism, consistent with many international code (Resnik et al., 2015; The Office of Research Integrity (ORI), 2022). Although these policies also conduct several other practices as misconduct or questionable practices, they differ in detail and are not yet clearly reflected in national policies. Inappropriate authorship, ghostwriting, multiple contribution, repetitive publication and published in different languages were specified in detail in a few universities, consistent with other study (Yi et al., 2019). Abuses, such as abuse of academic reputation, abuse of academic power, disclosure of academic secrets, and misuse of research resources, are not readily identifiable in many universities. These irresponsible behaviors' high occurrence and proportionally adverse effects are much more serious than those of FFP and deserve the attention of all kinds of RI management organizations in China.

### 4.2. Inquiry and investigation

We showed that inquiry and investigation, including Research Misconduct Reception and Investigation (RMRI), Sanctions for Research Misconduct (SRM), and Definition of Research Misconduct (DRM) are relatively fewer in number than the content of policies focused on the Definition of Research Integrity (DRI). The general procedure of investigation and its content detail is presented by Yi's study, they summarized that some universities set a pre-evaluation step for assessing the need of a formal investigation, with the faculty head or by a preliminary. Afterward, the formal investigation would be conducted by a specific/academic committee coordinated with other functional departments. In the end, behavior identification and treatment decision would be made by the special committee who was responsible for the investigation or the higher university organization (Yi et al., 2019). Others have reported that issues related to the inquiry and investigation include appointing the inquiry or investigation committee; conducting the inquiry or investigation; the contents of the inquiry or investigation report; and, who makes the decision on whether an investigation is warranted/ misconduct occurred (CHPS Consulting, 2000). In Japan, MEXT conducted a survey on university implementation of the “Guidelines for Responding to Misconduct in Research,” organized a special survey every year, and published the results (Ministry of Education, Culture, Sports, Science and Technology Japan, 2022). The survey inquired in great detail about the university's investigation procedure and committee, initiatives to raise awareness of research ethics, preservation and presentation of research data, and promotion of RI, especially novel and distinctive practices (Nouchi et al., 2020). It also requested that respondents provide evidence such as documents, protocols, names of meetings where decisions were made, and dates of resolutions. It seems the inquiry and investigation procedures and responsible members between China and other countries are similar, but there is still room for improvement to be more standardized, completed, and detailed in China.

### 4.3. Collective responsibility

The extent of the research misconduct policies at elite universities is variable in terms of their content, the majority of our 50 sampled universities emphasized the importance of the faculty and college-level supervision in preventing research misconduct and fostering RI, especially their role in promoting integrity and academic moral education, developing integrity policy, and assessing the quality and reliability of research results. Some studies reported that no policy reviewed specifically listed penalties for failure to report observed scientific misconduct (CHPS Consulting, 2000). Others reported the respondent researcher should be punished along with the academic promoter (for postgraduates) (Yi et al., 2019). In Japan, the whole faculty in which the researcher studied/worked maybe faced punishment as well (Ministry of Education, Culture, Sports, Science and Technology Japan, 2022). On the other hand, according to the UK government, universities have pledged to end the use of non-disclosure agreements NDAs (GOV.UK, 2022). NDAs are not only settlement agreements used in the UK but are also carried out at the University of Texas at Arlington (2023). Tanaka's study points out that there is a potential risk and possibility for universities to digest scandals within the organization as a way to avoid punishment (Tanaka, 2018). This view is thought-provoking and places a higher demand on the improvement of the policy establishment.

### 4.4. Ensuring a fair and appropriate investigation

In order to ensure a fair and appropriate investigation organizations must address three topics in their policies: maintaining confidentiality; avoiding conflicts of interest; and, ensuring appropriate expertise is available for the inquiry and investigation (CHPS Consulting, 2000). Our result provides evidence that all 50 sampled universities have formed relevant organizations responsible for the treatment of research misconduct and RI. These special organizations or working groups, overseeing the development of the allover inquiry and investigation processes, are required to consist of a limited number of researchers by national guidance (Ministry of Education of the People's Republic of China, 2014). Most of our sampled universities' policies utilized a number of measures to protect against conflicts of interest including the use of outside experts, limited terms for committee membership, excluding members of the same organizational unit from inquiry or investigation committees, and/or the use of signed statements for self-disclosure of possible conflicts, and it is consistent with other study (CHPS Consulting, 2000; Nouchi et al., 2020).

### 4.5. Respondent and whistleblower rights

No reviewed policies state any rights of respondent and whistleblower. To date, respondent and whistleblower rights are not yet clearly reflected in national policies. Just one university mentioned that anonymous reports should also be recorded and responded to proactively. Respondent rights most often stated in policies include the right to comment on the inquiry report, the right to comment on the investigation report, and various rights to notification related to the inquiry and investigation (CHPS Consulting, 2000; Ministry of Education, Culture, Sports, Science and Technology Japan, 2022). The five rights that are most often granted to whistleblowers in the policies are the right to notification related to the investigation, the right to be interviewed by the inquiry and/or investigation committee, and the right to review and comment on his/her interview summary, the right to comment on the investigation report and the right to notification related to the inquiry.

### 4.6. Reporting and pursuit of allegations

No reviewed policies state that members of the colleges and universities were obligated to report research misconduct. No reviewed policy specifically listed penalties for failure to report observed scientific misconduct. Regarding anonymous allegations, most policies did not specify whether this type of allegation would be pursued. These findings are also consistent with an international study (CHPS Consulting, 2000). Here, we find some practices that may serve as a reference point. In the UK, universities and research institutions that sign the “Concordat to Support Research Integrity” are required to prepare a short annual statement, including measures and activities used in RI education and practice, investigations of research misconduct cases, and a description of lessons learned from these cases that must be publicly released after approval by the unit's management (Universities UK, 2019).

There are some critical issues underlying the challenges faced by the evaluation and reward systems which are intimately bound up with RI fostering in China. Chinese universities apply the monetary reward incentive to promote scientific publication productivity for about 20 years, which lead to a radical increase in China's international scholarly publication (Shu, 2017). At the time, publications bring scholars cash rewards, the possibility of funding acceptance, and getting a promotion, which reveals the golden rule of academia in China: “SCI Supremacy” (Shu, 2017; Ke, 2021). However, tying publication requirements to those rewards can lure researchers into inappropriate behaviors (Tang, 2019), which outstripped the country's ability to promote rigor and curb academic misconduct (Tang, 2019). The boom of “SCI Supremacy” has distorted the whole direction of scientific research, and the reform of scientific research evaluation and reward system and thus prompting RI was imminent. China's national government departments confronted the situation and have introduced a series of reform efforts including the reform of the higher education evaluation and reward system. In 2016, “Opinions on Deepening the Reform of the System and Mechanism for Talent Development” (The Central Committee of the Communist Party of China, 2016) first proposed “Overcome the orientations of “Academic Credentials Only,” “Professional Titles Only,” “Papers Only,” and paper is not regarded as the restrictive condition to evaluate the application-oriented talents.” It was against “SCI Supremacy” and caused strong repercussions in Chinese academic community. In October 2020, The Central Committee of the Communist Party and the State Council of China issued “The Master Plan to Deepen the Education Evaluation Reform in the New Era” (The Central Committee of the Communist Party the State Council of China, 2020), which put forward the reform goals for China's education field, including improving the evaluation and award system of universities and colleges. Inheriting the previous policies, it proposed that “effectively address the deep-rooted problem “Five Only” (Scores Only, Enrollments Only, Academic Credentials Only, Papers Only, Titles Only),” “promote the classified evaluation of higher institutions, guide the scientific orientation of different types of universities, and establish their characteristics and levels.” The so-called “classified evaluation” is to adopt different evaluation systems according to different disciplines and industries (Ke, 2021).

The reform of the scientific research evaluation and reward system closely relates to the foresting of RI. It is the baton of scientific research development, allowing scientists to do their work well and universities to develop their education function and social service functions. There are still some problems after the reconstruction of the scientific research evaluation and reward system. One aspect is about global common issues. The scientific research evaluation and reward system is newly constructed and need time to adapt for most countries with young research integrity systems. The other aspect is the Chinese situation. China's scientific research system is a government-led national system, which is quite different from most countries. To build a localized scientific research evaluation and reward system, universities and colleges need guidance from authoritative institutions, as well as fair, just, and open data sources (Ke, 2021). First, the understanding of “breaking the five only” is not uniform within the universities, and a considerable number of scholars equate “not only” with “don't count” and “don't regard.” Some universities do not grasp the extent to which they should be implemented and suspend the performance awards for the year (Song et al., 2021). Second, for universities that mainly focus on basic research, scientific publications are the main form of output, and how to evaluate their output will become the big focus of reform under the requirement of breaking the “SCI supremacy” (Song et al., 2021). Third, the combination of qualitative and quantitative indicators has always been the evaluation method advocated by the science and technology communities in China, but in practice, the weights of qualitative and quantitative indicators are not balanced. Some universities even use quantitative indicators to replace expert evaluation in project and/or talent evaluation. Quantitative evaluation is an important basis for qualitative evaluation, and scientometric indexes enable experts to acquire enough information and have more adequate opinions, and their conclusions are more authoritative at a higher level of information integration. However, research quality cannot be measured simply by indicators, and peer review is a necessary and fundamental evaluation method that cannot be bypassed. The current over-reliance on hard indicators in science and technology evaluation is partly due to concerns about human relations in expert evaluation, and partly because administrators are used to quantitative management, indicating the “simplicity” of management thinking (Song et al., 2021). Nowadays, the reform of China's evaluation and reward system is in line with international standards, which is a major trend. For example, the time of evaluation cycle of UK REF (REF 2021, 2022) and Australia ERA (Australian Government, 2022) is relatively long, and China is extending the cycle time too.

This study has several limitations. First, the investigation procedures for handling research misconduct are not compared in detail. We are attempting to compare the amount and frequency of theme words from the perspective view of descriptive statistical analysis. Second, considering that there is a large number of universities in China, the results from this study cannot be generalized to all universities and all kinds of special colleges. A study of RI policies in China found that a total of 122 counts of misconduct were listed in the policies of 32 top Chinese universities (Yuan, 2011). Therefore, we chose the 50 elite universities, but they cannot simply represent all universities and colleges' status in China. Unfortunately, we only analyzed the contents of RI management policies and documents and did not sort out the evaluation and reward policies and documents to determine whether there are still universities that offer monetary rewards for scientific publications. We have to point out that following our search and clustering strategy, we did not find any sampled universities' policies and documents that mentioned monetary incentives for scientific publications. A final limitation centers on limited evidence of whether sampled universities and colleges have acted on the policies, such as how many research integrities cases have been reviewed and what the sanctions were which limits what can be concluded in how effective the policies and procedure are. Whether universities and colleges comply with their policies requires research through the process of investigating and handling research misconduct cases, which is a direction for future research.

Although most of the sample universities have developed a basic framework for regulations on the treatment of RI to instruct researchers and members of RI committees, the overall RI policies of these universities are still not as comprehensive as they could be. One university still does not have a university-level document dealing with scientific research; thus they fail to provide guidance in their policies on certain misconduct scenarios; these loopholes become hidden risks that may allow some personnel guilty of research misconduct to escape punishment. According to the analysis of the constitutions of the sample university, the weak links are the unstandardized procedures for accepting and investigating research misconduct, and the inconsistent channels for supervision and reporting. And the policy gaps are the rights of respondent/whistleblower, and allegations for reporting/pursuit. By analyzing the rules and regulations of universities' RI management offices, we find that domestic universities have initially formed RI management mechanisms in which academic committees coordinate RI work and special committees on RI play an active facilitating role. However, we also found policy gaps and urgent problems that need to be solved, and hope that our findings can support the formulation of policies. After this review study, we do believe that some improvements can be made.

## 5. Conclusions

The aim of the present research was to examine how Chinese elite universities' RI-management organization supervises and regulates themselves, and whether they have responded to national RI implementation and development in China. The findings will be of interest to summarize the effectiveness and shortcomings of RI management in China's elite universities and to provide a decision basis and policy reference for further strengthening the implementation of RI in China. Our findings reveal that the sample universities have developed their own RI management policies, set up RI management organizations, and promulgated institutional charters. Most universities have definitions of research misconduct which means they agree that this is something that should be regulated, but then when we take a closer look into these definitions, we see that they differ, as well as that these policies differ in what behavior is considered to be research misconduct. Some universities have engaged in even more detailed management by clarifying the authority and responsibilities of management organizations and refining the management process. The findings of this study suggest that Chinese elite universities have responded actively to the national call and issued their policies. Some limitations exist in this study. The small sample size did not allow it to be generalized to all universities and all kinds of special colleges in China. And it is unfortunate that the study did not include a detailed specific content analysis. We hope that future research could collect more data and overcome such limitations. Although this study presents a descriptive statistical analysis with visualization of the overall situation and main characteristics of RI regulations, we did not investigate how such regulations would influence research assessment and the reward systems of science in China, which could be explored by further research. In conclusion, it is still necessary to further strengthen the groundwork, optimize the workflow, implement the management responsibilities of RI weak links, and fill the gaps to achieve thorough RI supervision.

## Data availability statement

The original contributions presented in the study are included in the article/Supplementary material, further inquiries can be directed to the corresponding author.

## Author contributions

All authors listed have made a substantial, direct, and intellectual contribution to the work and approved it for publication.

## Supplementary material

Supplementary Table 1A The list of university's Policy Name, Year, Policy Subject Word Annotation, Type of Policy Theme, Definition of Research Misconduct or Irresponsible Behavior, the Policy Indicate that “Teachers Should be Jointly and Severally Liable and Punished for Misbehaving Students Due to Lack of Supervision” or Not, and their weblinks.

Supplementary Table 1B The information list of university's RI management organization for name (Chinses/English), its Superior Management Unit, title of the Management Organizations' Policies (Chinese/English) and Announcement of Investigation and Punishment Results.
